# Supplementary material for: Enhanced future changes in wet and dry extremes over Africa at convection-permitting scale
Source: Nat Commun. 2019 Apr 23;10:1794. doi: 10.1038/s41467-019-09776-9 (PMC6478940; doi:10.1038/s41467-019-09776-9)
Supplement: Supplementary file 1 — Supplementary Information [file 41467_2019_9776_MOESM1_ESM.pdf]

Enhanced future changes in wet and dry extremes over Africa at convection-permitting scale

Kendon et al.

Supplementary figures

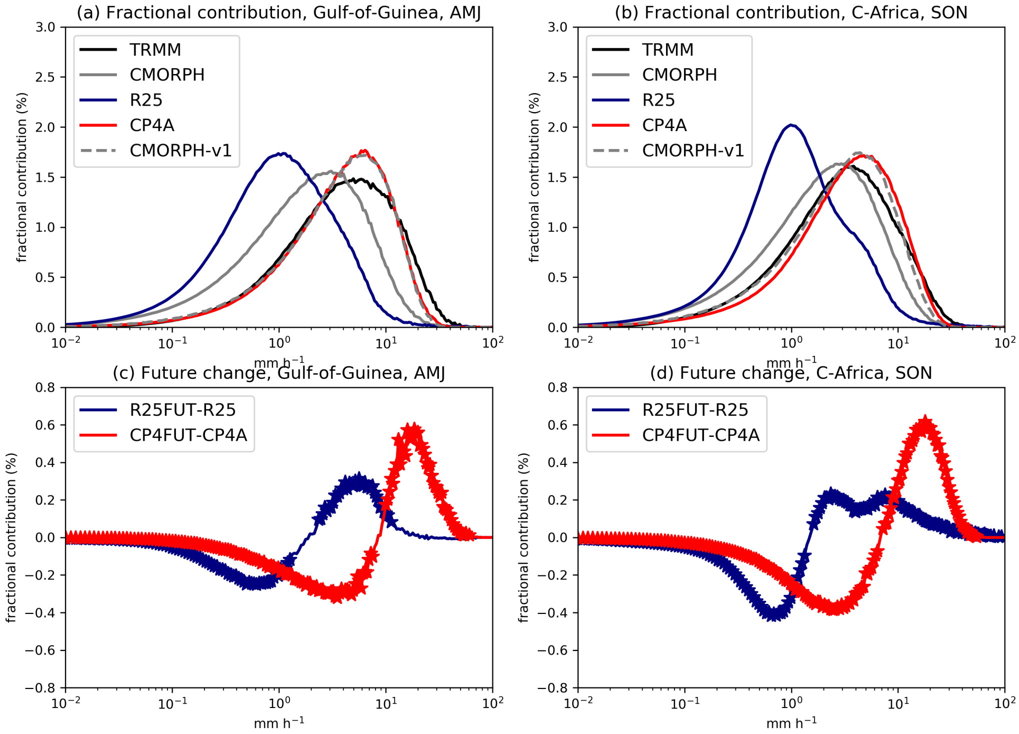

Supplementary Figure 1: Fractional contribution of 3-hourly precipitation intensity bins to the total precipitation. (a,b) Fractional contribution (%) for present-day for TRMM and CMORPH observations and R25 and CP4A models; (c,d) difference in fractional contribution between 2100 and present-day for R25 and CP4A models, for Gulf-of-Guinea April-May-June (AMJ) and C-Africa September-October-November (SON). For CMORPH, the original version 1 of the data (CMORPH-v1, grey dashed) is shown as well as the bias-corrected data (CMORPH, grey solid). All 3-hourly data in the given season, in the 10 year period, from all land points in the sub-region are used to calculate the fractional contribution. Stars indicate where future changes are significant at the 1% level compared to year-to-year variability, assessed using bootstrap resampling.

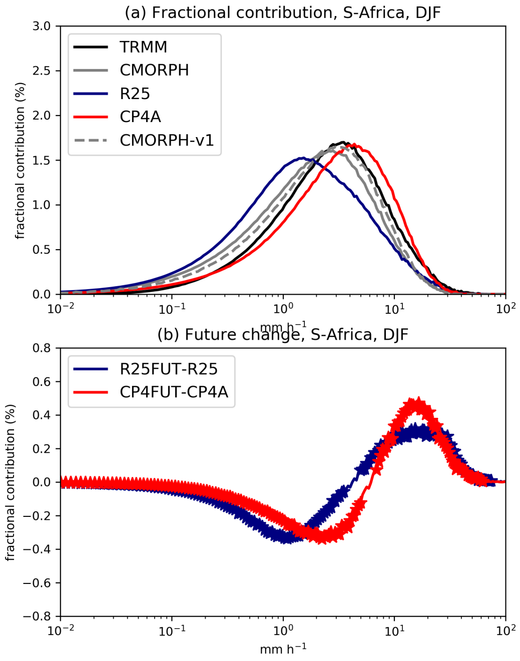

Supplementary Figure 2: As Supplementary Figure 1, but for S-Africa December-January-February (DJF).

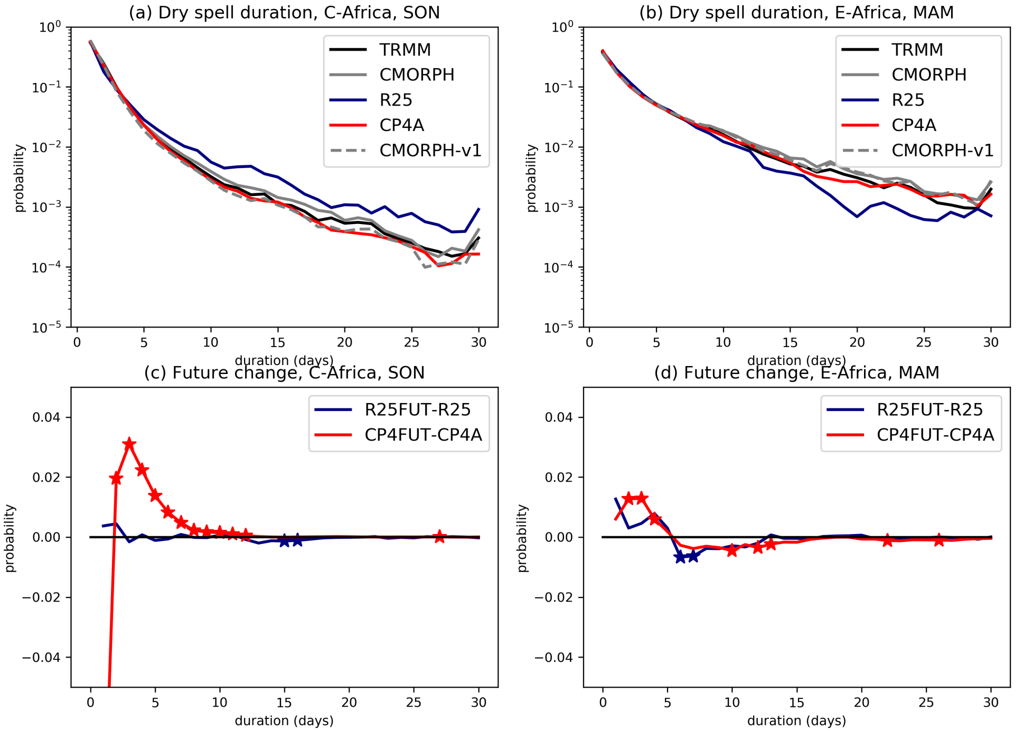

Supplementary Figure 3: Probability distribution of dry spell duration. (a,b) Present-day duration of dry spells for TRMM and CMORPH observations and R25 and CP4A models; (c,d) differences in dry spell distribution between 2100 and present-day for R25 and CP4A models, for C-Africa September-October-November (SON) and E-Africa March-April-May (MAM). Dry spells are defined as days with <1mm of rainfall. For CMORPH, the original version 1 of the data (CMORPH-v1, grey dashed) is shown as well as the bias-corrected data (CMORPH, grey solid). All daily data in the given season, in the 10 year period, from all land points in the sub-region are used to calculate the distribution. Stars indicate where future changes are significant at the 1% level compared to year-to-year variability, assessed using bootstrap resampling.

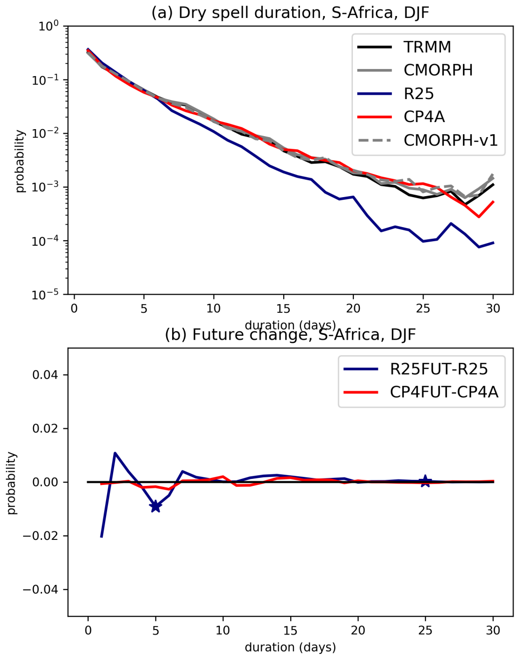

Supplementary Figure 4: As Supplementary Figure 3 but for S-Africa December-January-February (DJF).

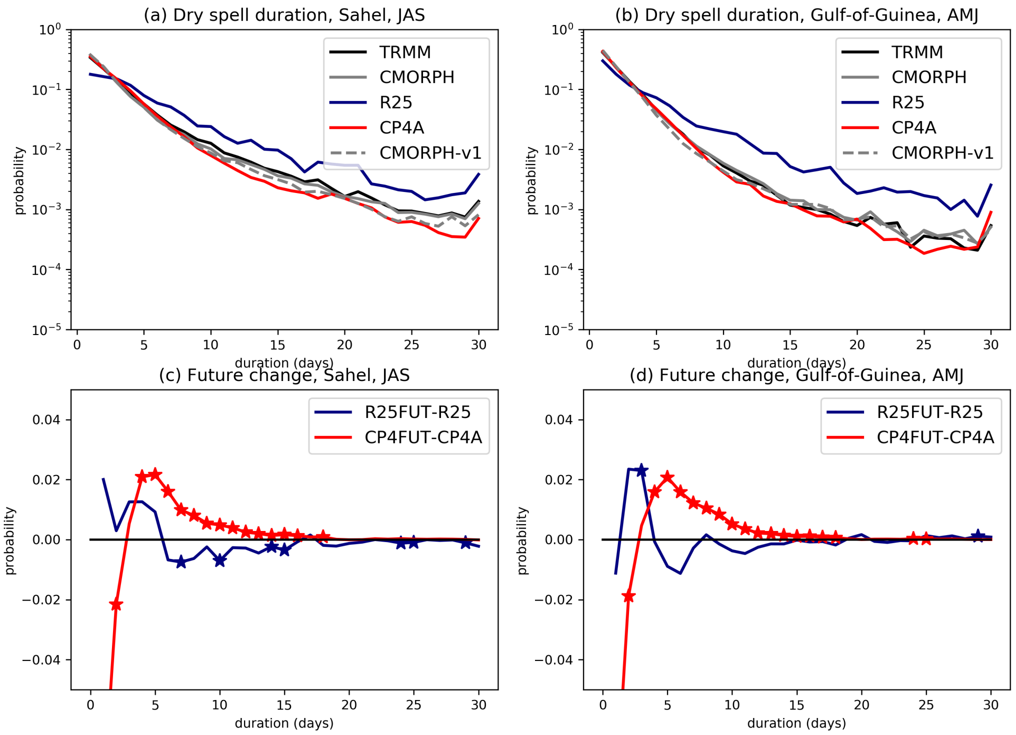

Supplementary Figure 5: As Supplementary Figure 3 but for Sahel July-August-September (JAS) and Gulf-of-Guinea April-May-June (AMJ), and using a percentile-based approach for defining a dry spell. Dry spells are defined as days with rainfall less than the present-day 65th percentile of wet season daily precipitation across all African grid points corresponding to 0.51 mm for CP4, 2.95 mm for R25, 0.59 mm for TRMM, 0.68 mm for CMORPH and 0.90 mm for CMORPH-v1.

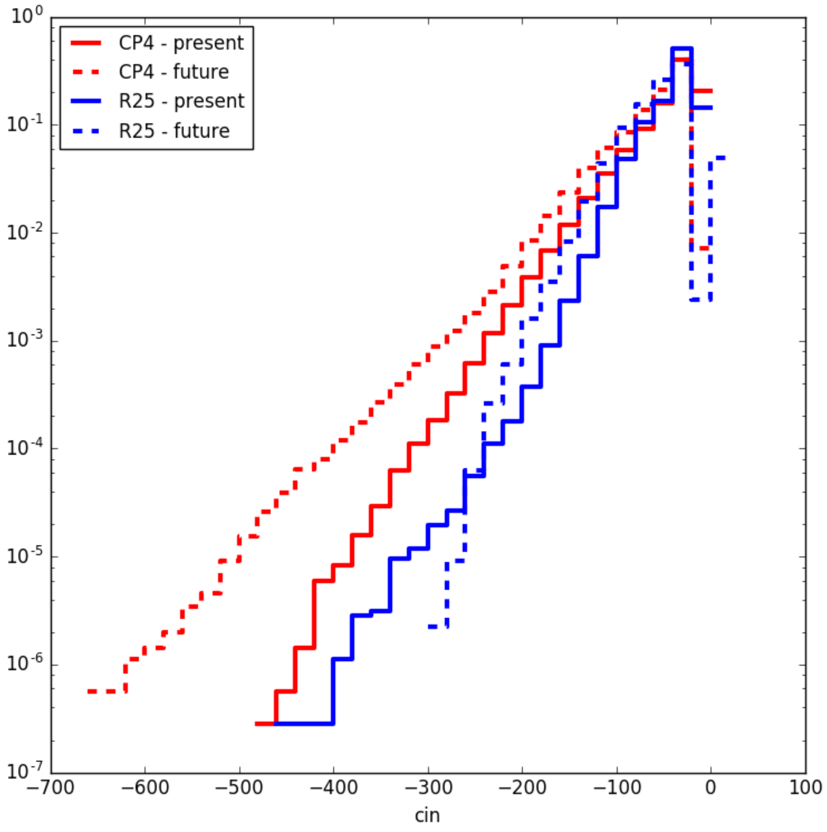

Supplementary Figure 6: Future changes in convective inhibition (CIN). Probability distribution of CIN (J kg^{-1}) for the region $5-15°$N, 10°W-10°E, for July-August-September (JAS) 2004, for CP4A and R25 present-day and future simulations. CIN is calculated from hourly atmospheric profiles by raising a parcel from 20m. In both models, under climate change, there is an decrease in frequency of occurrence of very small CIN values and an increase in frequency of occurrence of larger (negative) CIN values, which will act to inhibit convective storms.

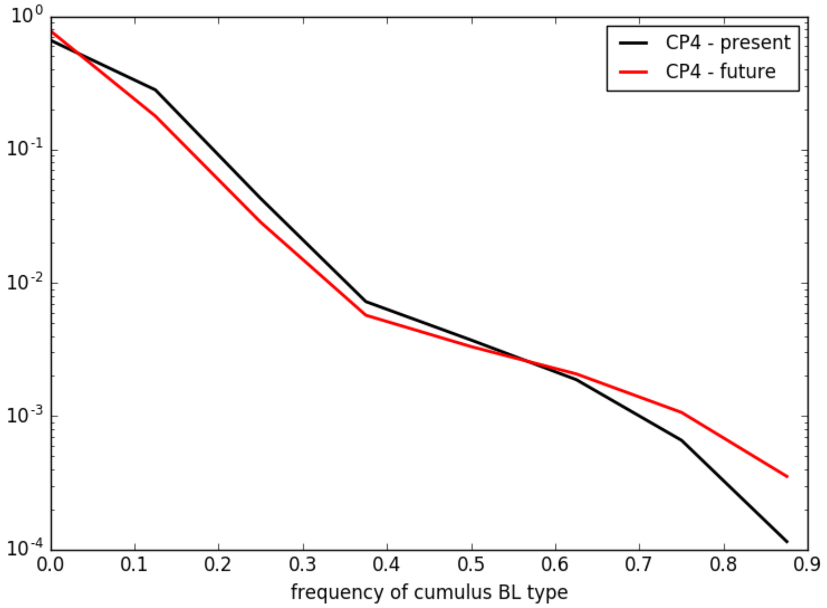

Supplementary Figure 7: Frequency of occurrence of cumulus boundary layer type. Probability distribution of daily mean frequency of occurrence of the cumulus boundary layer type, for the region $5-15°$N, 10°W-10°E, for July-August-September (JAS) 1997-2000, for CP4A present-day and future simulations. The diagnosis of a cumulus boundary layer type indicates that the atmospheric profile at that time is able to support convection from the surface layer. The number of cumulus boundary layer types is decreasing in the future climate (more values at 0, fewer between 0.1-0.6), except in the very small number of cases where cumulus is being diagnosed most of the day.

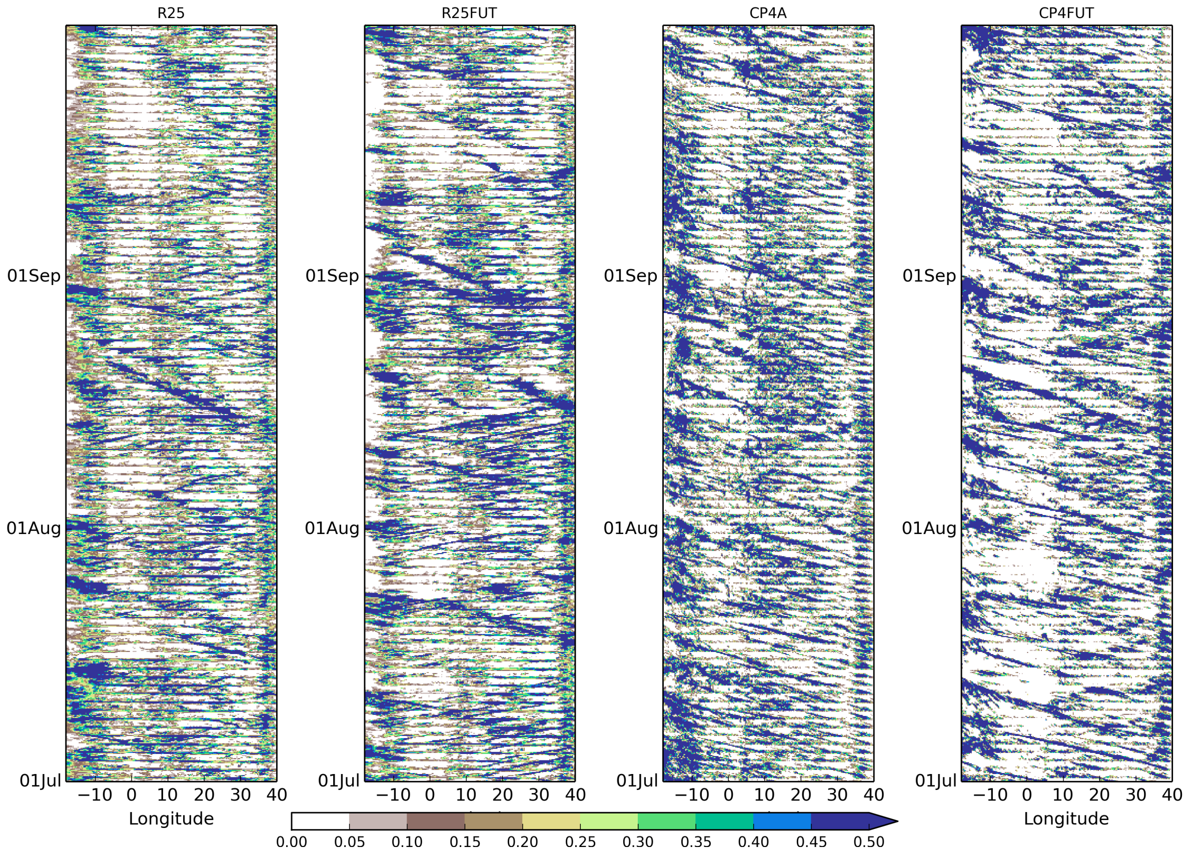

Supplementary Figure 8: Hovmöller plots of 3-hourly precipitation. Precipitation (mm h^{-1}) averaged over the latitude band $5 - 15°$N for July-August-September (JAS) 1998 and for the equivalent season in the future simulation, for R25 and CP4A.

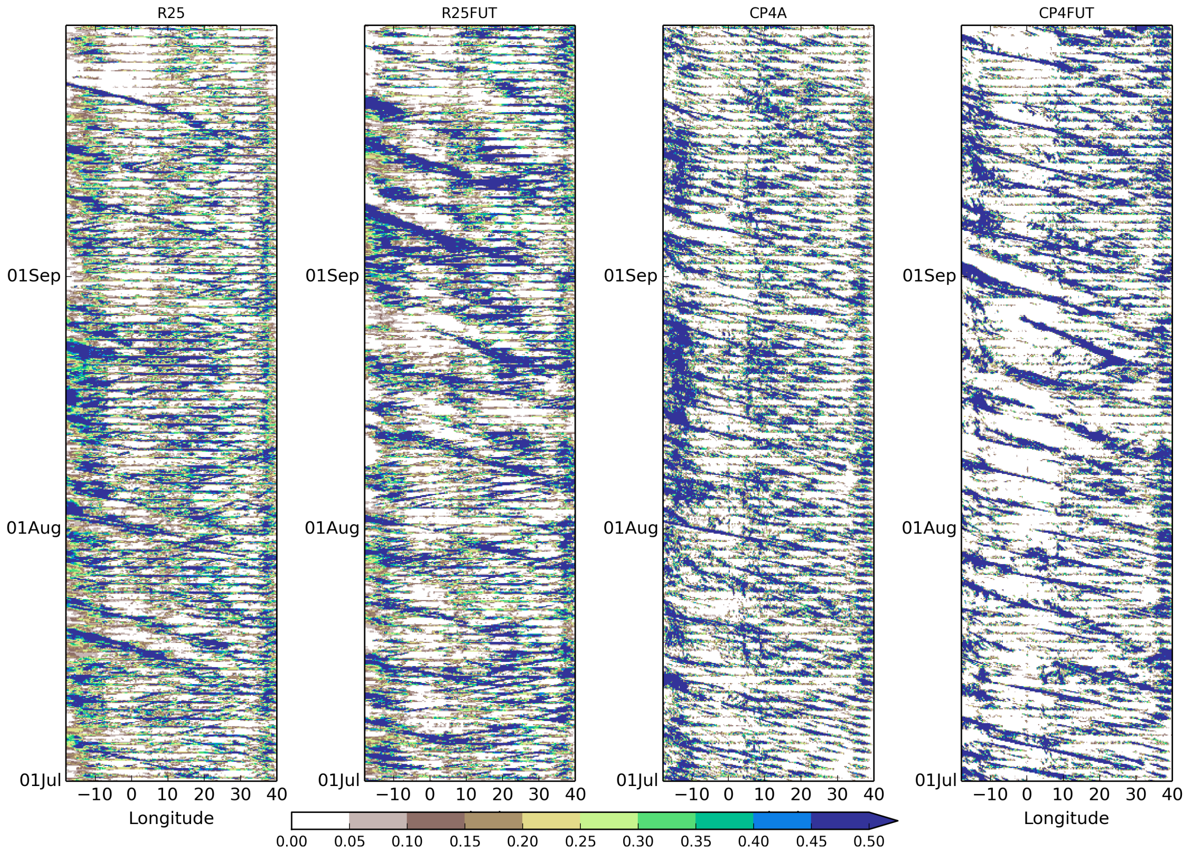

Supplementary Figure 9: As Supplementary Figure 8 but for JAS 2001.

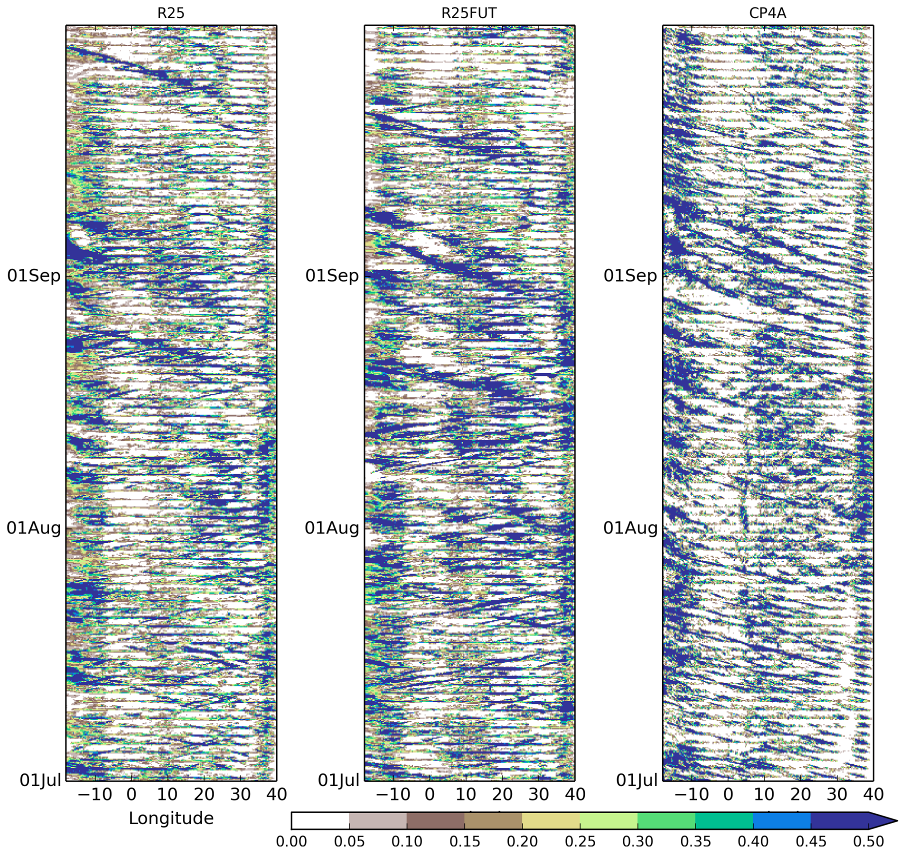

Supplementary Figure 10: As Supplementary Figure 8 but for JAS 2004.

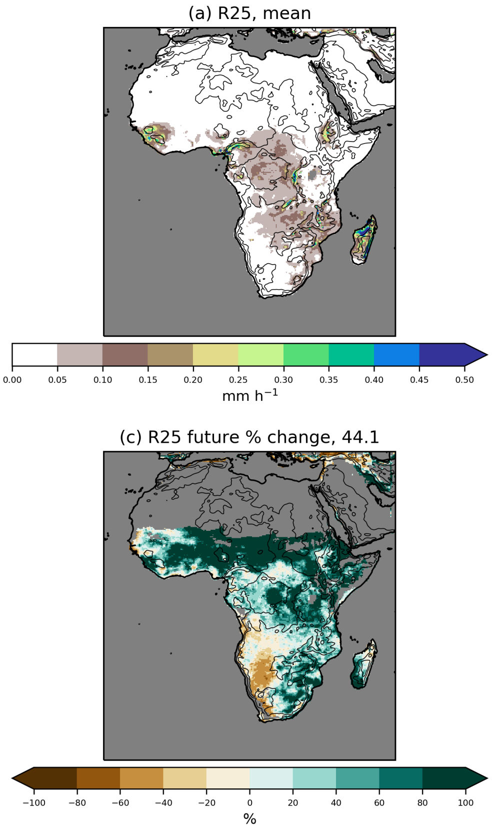

Supplementary Figure 11: Total mean runoff during the wet season. (a,b) Present-day runoff and (c,d) percentage differences between 2100 and present-day, for R25 and CP4A models. The median of future percentage changes across Africa is indicated in the panel titles. Future changes are masked in grey where present-day runoff is less than 0.01 mm h^{-1}. The wet season is the 3 month period with the highest mean precipitation in TRMM, defined on a grid-point basis. The black lines indicate the 500m, 1000m, 2000m, 3000m and 4000m height contours.

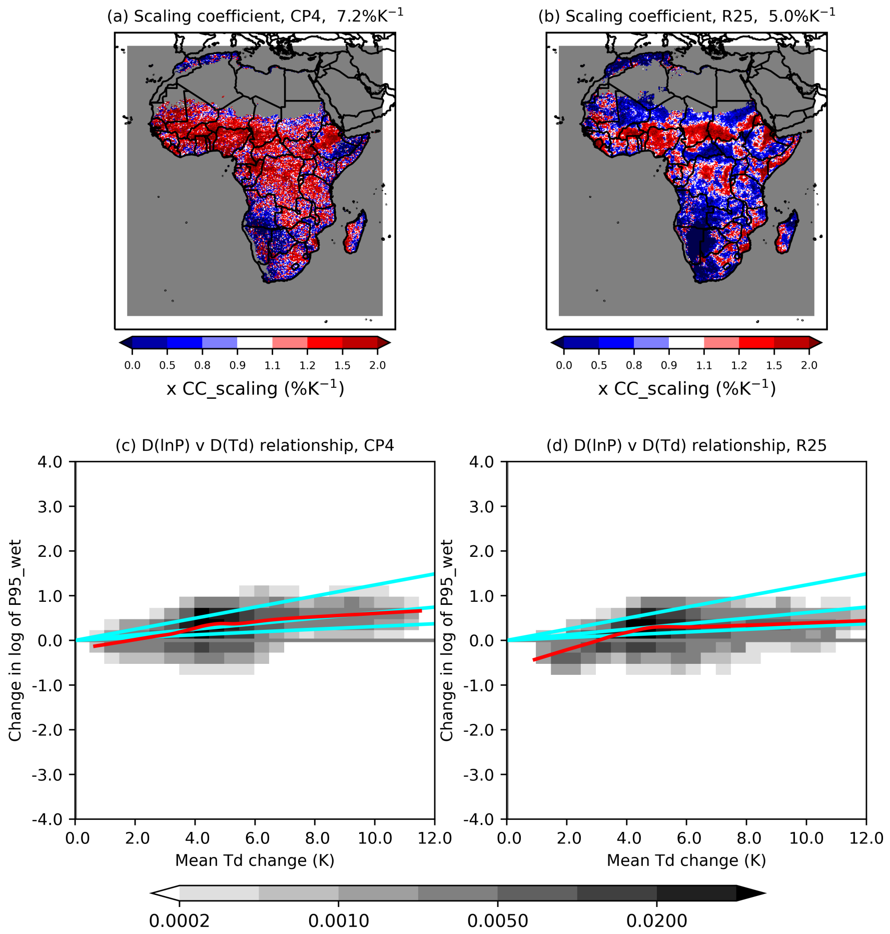

Supplementary Figure 12: Scaling between future changes in extreme daily precipitation intensity and dew point temperature. (a,b) Scaling coefficient given by future change in logarithm of extreme daily precipitation intensity divided by future change in mean dew point temperature, for the wet season, for CP4A and R25. The median scaling across Africa is indicated in the panel titles; colours correspond to the scaling coefficient divided by the Clausius-Clapeyron relationship of 6.2%K^{-1} (such that a value of 1 corresponds to CC-scaling). (c,d) Joint probability distribution of change in logarithm of extreme precipitation intensity versus change in mean dew point temperature T_{d}, for the wet season, across Africa, for CP4A and R25. Cyan lines show the relationship for 0.5, 1 and 2 times CC-scaling; red lines show the average relationship obtained from fitting a Lowess regression line. Extreme daily precipitation intensity is defined as the 95th percentile of wet days (>1mm day^{-1}), for daily precipitation, and is set to missing (and masked in grey) where less than 5% of the data is wet. The wet season is the 3 month period with the highest mean precipitation in TRMM, defined on a grid-point basis.

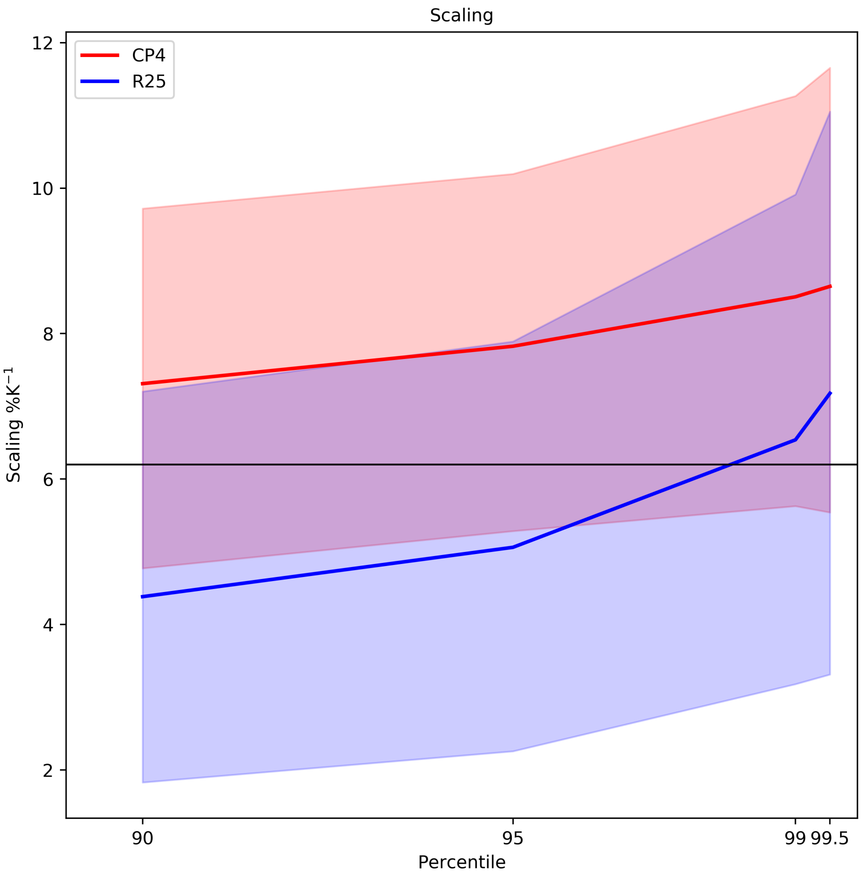

Supplementary Figure 13: Scaling rate across Africa (%K^{-1}) as a function of wet-value percentile. Plotted is the median scaling rate across Africa, with the shaded region showing the interquartile range in spatially-varying scaling rates. Results are shown for percentiles of daily maximum 3-hourly precipitation, for wet values (>0.1mm h^{-1}) only. Scaling at each grid point is given by the future change in the logarithm of precipitation, for the given percentile, divided by future change in mean dew point temperature, for the wet season locally. Local values are masked where less than 5% of the data is wet. The Clausius-Clapeyron relationship of 6.2%K^{-1} is shown in black.

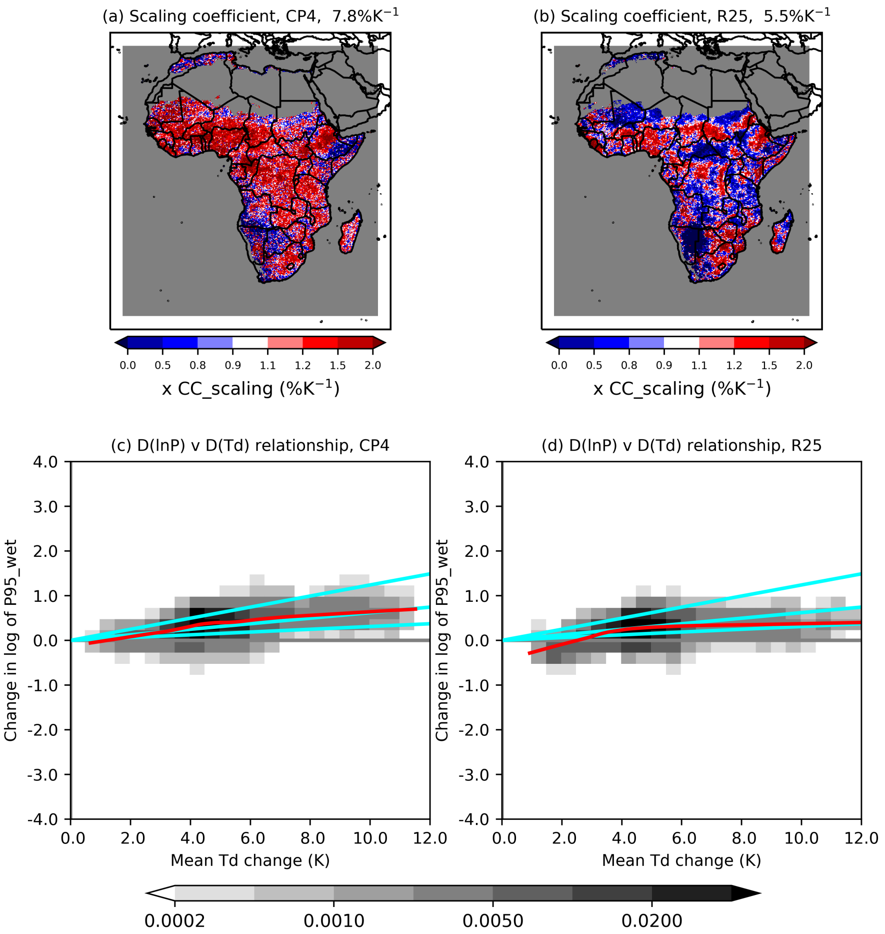

Supplementary Figure 14: As Supplementary Figure 12 but for scaling between future changes in extreme 3-hourly precipitation intensity and dew point temperature, and using a percentile-based approach for defining a wet value. Extreme precipitation intensity is defined as the 95th percentile of wet values, for daily maximum 3-hourly precipitation; where a wet value is defined as rainfall greater than the present-day 65th percentile of wet season daily maximum 3-hourly precipitation across all African grid points corresponding to 0.129 mm h^{-1} for CP4 and 0.533 mm h^{-1} for R25. Using a percentile-based threshold for wet values ensures the same number of extreme events are selected from each model in the present-day.

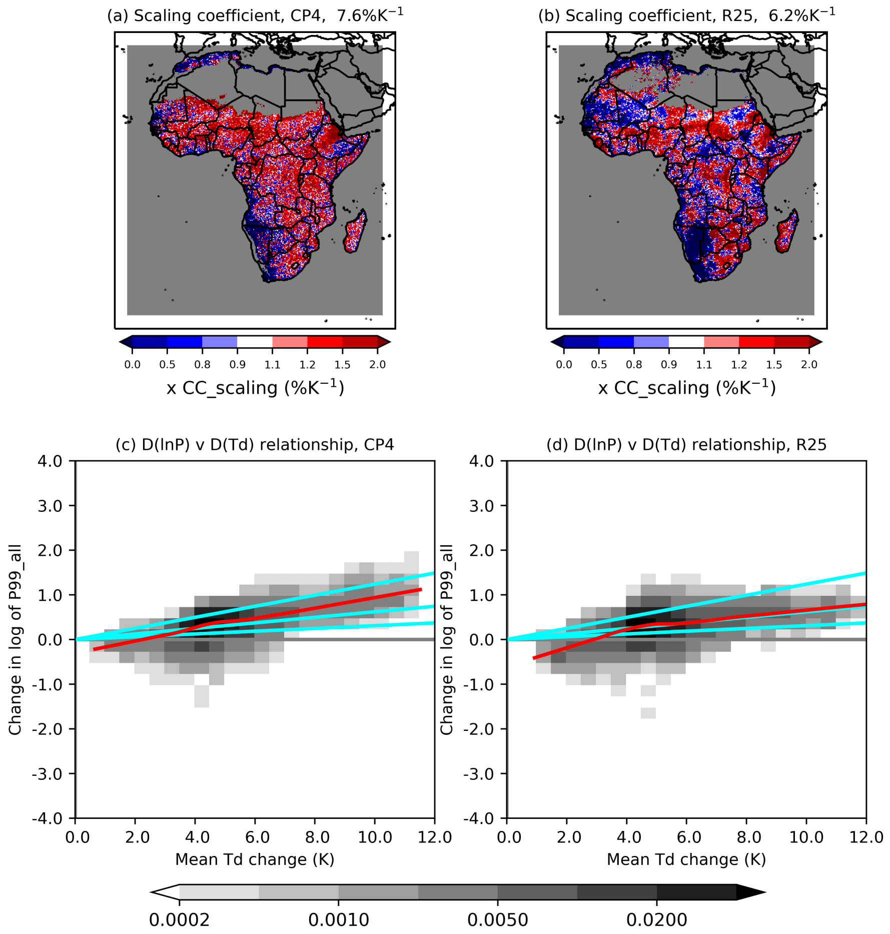

Supplementary Figure 15: As Supplementary Figure 12 but for scaling between future changes in extreme 3-hourly precipitation and dew point temperature. Extreme precipitation is defined as the 99th percentile of all values, for daily maximum 3-hourly precipitation.

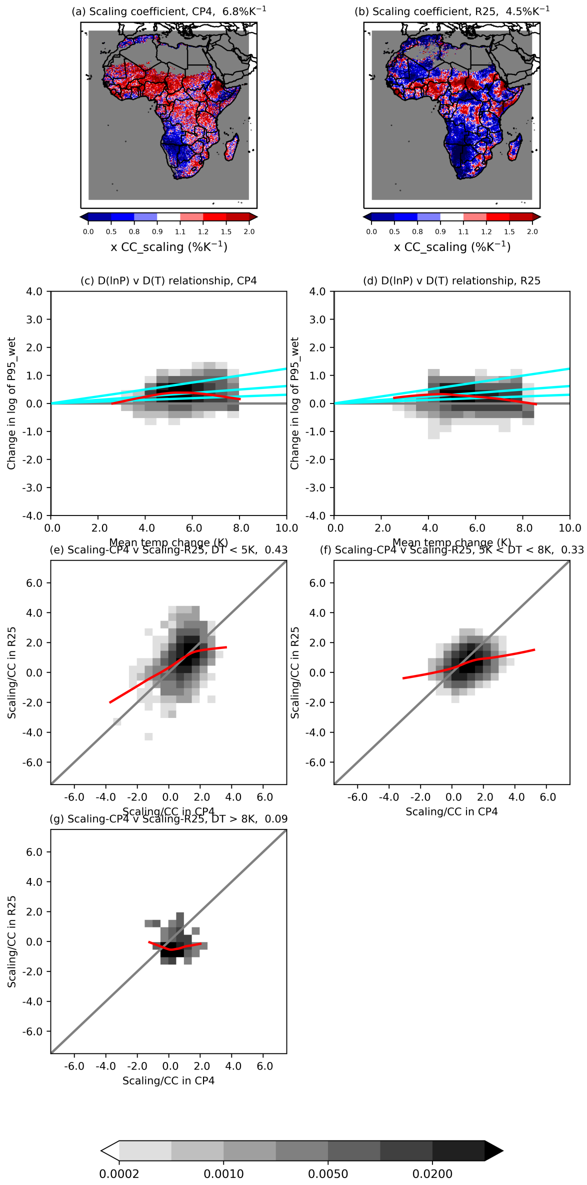

Supplementary Figure 16: As Supplementary Figure 12 but for scaling between future changes in extreme 3-hourly precipitation intensity and 1.5m temperature. Also shown is joint probability distribution of scaling coefficient in CP4A versus scaling coefficient in R25, for mean temperature change of (e) <5K, (f) 5< $\triangle T$ <8K and (g) >8K. The correlation between CP4A and R25 scaling coefficients is indicated in the panel titles. Extreme precipitation intensity is defined as the 95th percentile of wet values (>0.1mm h^{-1}), for daily maximum 3-hourly precipitation.
